# Maternal lipid profile and risk of pre-eclampsia in African pregnant women: A systematic review and meta-analysis

**DOI:** 10.1371/journal.pone.0243538

**Published:** 2020-12-23

**Authors:** Endalamaw Tesfa, Endalkachew Nibret, Abaineh Munshea

**Affiliations:** 1 Department of Biochemistry, College of Medicine and Health Sciences, Bahir Dar University, Bahir Dar, Ethiopia; 2 Biotechnology Research Institute, Bahir Dar University, Bahir Dar, Ethiopia; 3 Department of Biology, College of Science, Bahir Dar University, Bahir Dar, Ethiopia; University of Mississippi Medical Center, UNITED STATES

## Abstract

**Introduction:**

Some studies have reported the association between maternal serum lipid profile abnormalities and pre-eclampsia. However, many studies have reported controversial results. Hence, this systematic review and meta-analysis was planned to generate summarized evidence on the association between maternal serum lipid profiles and pre-eclampsia in African women.

**Methods:**

Four electronic databases such as; PubMed, Hinari, Google Scholar, and African Journals Online were searched for studies published in English. Joanna Briggs Institute Meta-Analysis of Statistics Assessment and Review Instrument and Newcastle-Ottawa Scale were used for data extraction and quality assessment of the included studies. The meta- regression analysis was performed by Stata 14 software. The standardized mean difference (SMD) values of lipid profiles were computed to assess their association with pre-eclampsia at 95% CI.

**Results:**

In this review a total of 15 observational studies were included. The mean values of triglyceride (TG), total cholesterol (TC), low density lipoprotein- cholesterol (LDL-c) and very low density lipoprotein- cholesterol (VLDL-c) were significantly higher in pre-eclamptic women as compared with normotensive pregnant women (TG = 229.61±88.27 and 147.00 ± 40.47, TC = 221.46 ± 45.90 and 189.67 ± 39.18, LDL = 133.92 ± 38.77 and 112.41 ± 36.08, VLDL = 41.44 ± 19.68 and 26.64 ± 7.87), respectively. The serum high density lipoprotein cholesterol (HDL-c) level was lower, but it is not statistically significant (HDL-c = 51.02 ± 16.01 and 61.80 ± 25.63) in pre-eclamptic women as compared with controls. The pooled standardized mean difference (SMD) of TG, TC, LDL-C and VLDL-C were significantly increased in pre-eclamptic women as compared with normotensive pregnant women with the SMD of (TG = 1.65 (1.10, 2.21), TC = 0.84 (0.40, 1.29), LDL-C = 0.95 (0.46, 1.45) and VLDL-C = 1.27 (0.72, 1.81)) at 95% CI, respectively, but the pooled SMD of HDL-cholesterol was decreased in pre-eclamptic women as compared with normotensive pregnant women (SMD = -0.91 (95% CI: -1.43, -0.39).

**Conclusions:**

In this review, the maternal serum levels of TG, TC, LDL-c and VLDL-c were significantly associated with the risk of preeclampsia. However, HDL- cholesterol was not significantly associated but it was lower in pre-eclamptic women. Further, large scale prospective studies should verify these outcomes and it is recommended that lipid profiles should be included as a routine diagnostic test for pre-eclamptic women.

## 1. Introduction

Pre-eclampsia is a pregnancy related metabolic syndrome characterized by hypertension and proteinuria occurred after 20 weeks of gestation. It is the most common cause of maternal and prenatal morbidity and mortality [1]. The cause of pre-eclampsia is not clearly known, but failure of spiral artery remodeling cause’s placental ischemia and maternal syndrome resulted in hypertension and proteinuria [2]. Increased in lipid oxidation products and decreased in the levels of antioxidants involved in the pathogenesis of pre-eclampsia [3]. Different reports showed that the risk of pre-eclampsia increases in the women with higher levels of oxidized low-density lipoprotein (LDL) and triglycerides (TG) and lower levels of circulating vitamin C as compared to normotensive pregnant women [4, 5]. The oxidative conversion of LDL- cholesterol to oxidized LDL form is the key event for the initiation and development of atherosclerosis and hypertension [4].

Early pregnancy dyslipidemia is associated with an increased risk of pre-eclampsia [1]. In the early pregnancy, the mother exists in the anabolic state and the lipid serves as a source of calories for the growing fetus as well as for the mother in the third trimester [6]. There are many facts that show lipid profile abnormalities might be associated with the risk of pre-eclampsia. Lipid profiles such as: TG, LDL-c, total cholesterol (TC) and very low density lipoprotein (VLDL) levels are higher in pre-eclamptic women as compared with normal pregnancies, but high-density lipoprotein (HDL) level is lower in pre-eclamptic women as compared with normal pregnancies [7–9]. Lipid profile abnormalities increases as the gestational age of the mother increases. A meta-analysis study consisted of 24 case control studies confirmed that there is a strong association between hypertriglyceridemia and the risk of pre-eclampsia [10].

Different studies have tried to elucidate the association between the maternal serum lipid profiles and pre-eclampsia, but many of them have reported conflicting results. Some studies have shown higher levels of lipid profiles in pre-eclamptic women as compared to normotensive pregnant women [9, 11–13]. However, a few studies have found non-significant difference in the serum levels of maternal lipid profiles between pre-eclamptic and normotensive pregnant women [14, 15] and few other reports indicated lower serum lipid profiles in pre-eclamptic women as compared to normotensive controls. Therefore, this systematic review and meta-analysis was designed to generate summarized evidence between maternal serum lipid profiles and risks of pre-eclampsia in African pregnant women.

## 2. Methods and materials

### 2.1. Protocol and registration

This review protocol is registered at the National Institute for Health Research; PROSPERO international prospective register of systematic reviews with registration number CRD42020192865 at (https://www.crd.york.ac.uk/prospero/#recordDetails).

### 2.2. Study design and search strategy

A systematic review and meta-analysis of published studies was conducted to assess the association between maternal serum lipid profiles and risk of pre-eclampsia in African pregnant women. We searched for the following databases: PubMed, Hinari, African Journals Online (AJOL) and Google Scholar. The search was done by using the following Medical Subject Heading (MeSH) terms; “Lipids, Triglycerides, Cholesterol, HDL, VLDL, LDL, Pre-eclampsia and Africa*”* separately or in combination by using string term Or AND. All published and unpublished articles up-to July 30, 2020 were retrieved and assessed for their eligibility for their inclusion in this review. Preferred Reporting Items for Systematic Reviews and Meta-Analyses (PRISMA) guideline was utilized to conduct this systematic review and meta-analysis.

### 2.3. Eligibility criteria

Inclusion and exclusion criteria;

1. Studies conducted on human subjects were included.
2. Studies with case-control, cross-sectional and cohort designs in Africa were included.
3. Articles that report pre-eclampsia as an outcome variable were included.
4. Published and unpublished articles written in English were included.
5. Studies considering lipid profiles as the determinant variable and reporting the result in mean and standard deviation were included.
6. Studies conducted in the community or in the health institution were included.
7. Conference papers, editorials, reviews and randomized control trials were excluded.

### 2.4. Study selection and screening

**Fig 1:**
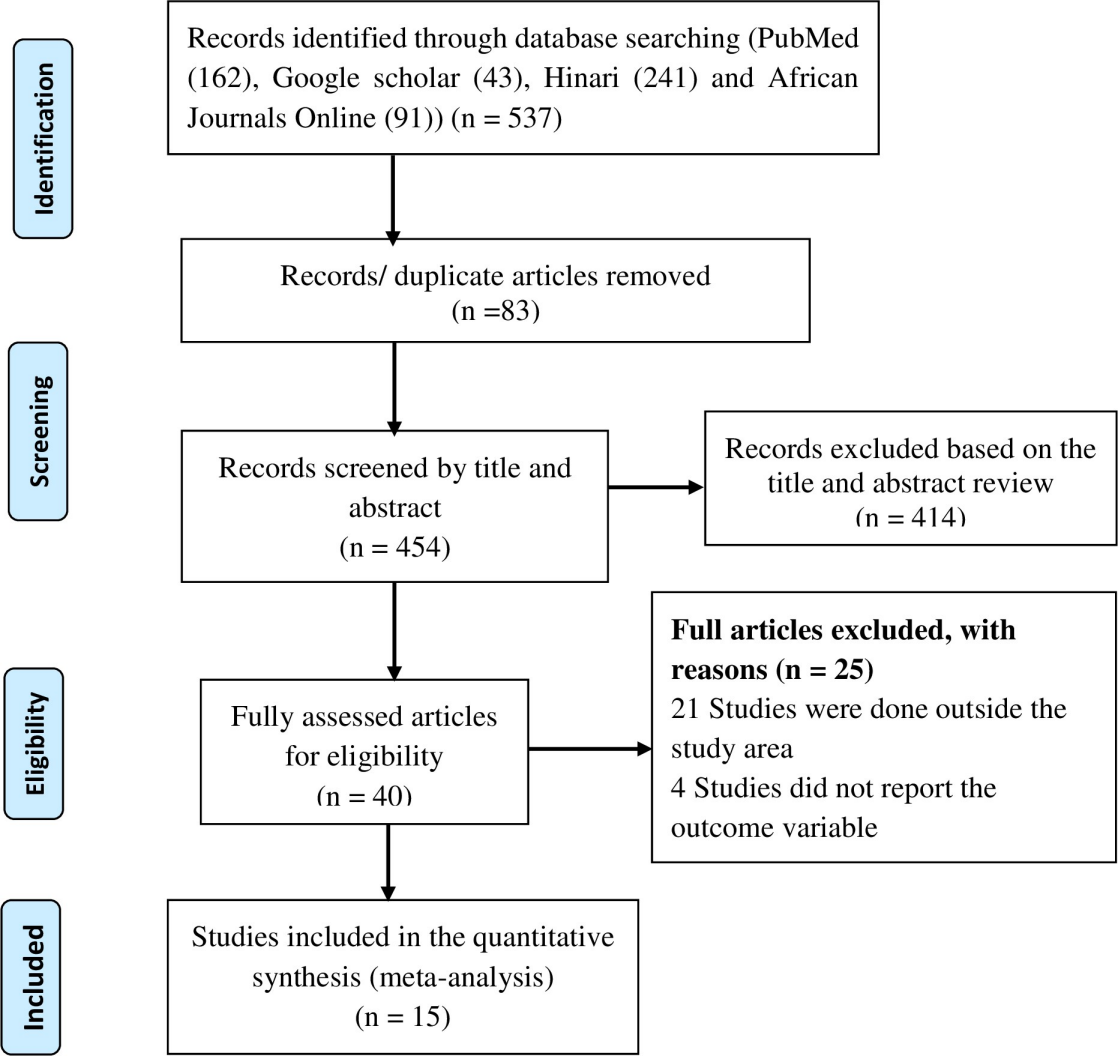
Flow diagram showing the included studies for the systematic review and meta-analysis of maternal lipid profile and pre-eclampsia.

All citations identified by our search strategy were exported to EndNote -X9- and duplicate articles were removed. And then the titles and abstracts of the identified articles were screened by two independent reviewers, and eligible studies were included for further review. The full texts of selected articles were retrieved and read thoroughly to ascertain the suitability prior to data extraction. In case of disagreement between the two reviewers, discussion was held to reach consensus and the third reviewer was consulted. The search process was presented in PRISMA flow chart that clearly shows the studies that were included and excluded with reasons of exclusion (Fig 1) [16].

### 2.5. Definition of outcome interest

The primary outcome of this study is to evaluate the association between maternal serum lipid profiles with the risk of pre-eclampsia in African pregnant women.

- Hypertension is defined as the systolic blood pressure ≥140mmHg and/or diastolic ≥90mmHg that are measured at least two times within four hours interval.
- Proteinuria: urinary protein excretion of ≥300mg /24-h urine sample or ≥1*+* on qualitative dipstick examination or a total protein: creatinine ratio ≥30 mg/ mmol (or ≥0.3 when both are measured in mg/dL).
- Gestational hypertension: hypertension diagnosed after 20 weeks of gestation.
- Pre-eclampsia is defined as hypertension plus proteinuria after 20 weeks of gestation.
- Eclampsia: Seizures in women with hypertension that cannot be attributed to other causes [17].

### 2.6. Quality assessment

For case-control and cohort studies, we used Newcastle-Ottawa Scale (NOS) to assess the quality of the included studies while for cross sectional studies the modified version of NOS was used to assess the quality of the studies for inclusion [18]. The NOS included 3 categorical criteria with a maximum score of 9 points. The quality of each study was rated using the following scoring algorithms: ≥7 points was considered as “Good”, 4 to 6 points was considered as “Fair”, and ≤3 point was considered as “Poor” quality study. In order to improve the validity of the review result, we only included primary studies with fair to good quality [18].

### 2.7. Data extraction process

The data extraction was done using a tool developed by the 2014 Joanna Briggs Institute Reviewers’ Manual data extraction form [19]. The abstract and full-text were reviewed by the two independent reviewers. Data extraction includes: author’s name, publication year, study country, study design, sample size, number of cases and controls, mean age, mean gestational age, mean BMI, mean SBP and DBP, mean TG, TC, HDL, LDL and VLDL levels were extracted. Standard error of mean (SEM) was computed by the formula SEM = SD/√*n*. Lipid profiles measured in mmol/L was converted into mg/dL by multiplying their conversion factors. For few studies that didn’t report VLDL value we estimated from TG level by dividing into five.

### 2.8. Data analysis

The data were entered into Microsoft excel and the meta-analysis was performed using Stata 14 software and SPSS version 20. Forest plot of standardized mean difference (SMD) were used to assess the effect size between the serum levels of lipid profiles and pre-eclampsia at 95% CI. The SMD is the ratio of the mean difference to the pooled standard deviation. Standard error of mean (SEM) was also computed by dividing standard deviation to the square root of the sample size. Subgroup analysis was done by country (Nigeria, Ghana and Egypt). Variables including; maternal age, gestational age, BMI, mean lipid profiles were assessed through paired sample test to know their difference among pre-eclamptic and normotensive pregnant women.

### 2.9. Heterogeneity and publication bias

Statistical heterogeneity was estimated through Cochrane Q, I^2^ statistic and P-value. If I^2^ statistic values < 25%, 25–50%, and ≥50% were used to declare the heterogeneity test as low, medium and high, respectively. In this review, a random effect model (REM) was used for analysis. To cope with the reasons of high heterogeneity subgroup analysis and sensitivity test were performed. Publication bias was assessed through funnel plot and Egger’s test.

## 3. Results

### 3.1. Study selection

A total of 537 articles were retrieved through electronic search by using different search terms of which 454 article were eligible for title and abstract assessment after removal of 83 duplicate records. Out of 454 articles screened for eligibility 414 records were excluded after assessing their title and abstract. A total of 40 articles underwent full- text assessment, 25 studies were excluded due to different reasons (21 articles were done outside the study area and four articles didn’t report outcome variable).

### 3.2. Study characteristics

In this review a total of 15 studies were included. Ten of them were case-control and three studies were Cross-sectional and the other two were cohort. Studies that have been conducted in Africa up- to July 30, 2020 were included. Ten studies were conducted in Nigeria and the other three and two studies were conducted in Ghana and Egypt respectively. In this review a total of 2,106 pregnant women were included (828 cases and 1, 273 controls) (Table 1).

**Table 1:** Characteristics of research articles included in the systematic review and meta-analysis (N = 15).

S. No	Authors & Publication year	Country	Sample Size	PE (n =)	NP (n =)	Study Design	TC PE mg/dL	TC NP mg/dL	TGPE mg/dL	TGNP mg/dL	HDL PE mg/dL	HDL NP mg/dL	LDL PE mg/dL	LDL NP mg/dL	VLDL PE mg/dL	VLDL NP mg/dL	Quality score
1	Ahmed et al., 2018 [12]	Eygpt	100	60	40	Case- control	223.24± 11.55	176.13± 8.09	202.95±14.24	152.3±9.22	38.22±5.8	43.28 ± 4.03	145.53 ± 11.63	102.13 ± 9.78	40.72 ± 2.97	30.53 ± 1.91	6 points
2	Ahenkorah et al., 2008 [20]	Ghana	80	30	50	Case- control	244.4± 11.6	247.87 ± 8.51	303.8±16.83	225±23.03	72.7±4.25	76.95 ± 3.09	171.7 ± 7.73	148.5 ± 7.35	19.72 ± 1.55	20.88 ± 1.93	7 points
3	Avidime et al., 2018 [21]	Nigeria	140	70	70	Cross-sectional	251.35± 62.65	239± 47.95	243.6±110.72	160.32±48.72	49.88±13.15	55.68 ± 18.18	143.85 ± 40.6	106.34 ± 36.35	21.27 ± 9.67	13.92 ± 4.25	7 points
4	Olalere et al., 2018 [22]	Nigeria	240	120	120	Case- control	309.9± 113.92	237± 74.49	203.3±120.49	157.5±77.77	63.2±27.39	55.4 ± 19.72	156.5 ± 120.49	109.7 ± 82.48	39.5 ± 21.91	31.5 ± 15.33	7 points
5	Njoku, 2017 [23]	Nigera	116	58	58	Case- control	217.85± 68.31	219.71 ± 58.26	213.99±101	140.96±49.38	44.97±19.88	46.34±15.41	135.69±66.19	130.62±53.79	42.8±20.21	28.25±9.82	7 points
6	Nwachuku, 2017 [24]	Nigeria	140	70	70	Cross-sectional	257.15± 83.14	176.33 ± 37.12	199.3±89.46	128.43±55.8	46.79±18.95	36.35± 11.6	172.85 ±75.8	109.05 ± 35.58	39.83 ± 17.79	25.52 ± 11.21	7 points
7	Yeboah et al., 2017 [14]	Ghana	312	26	286	Cohort	232± 65.74	224.3 ± 69.6	139.95±70.86	142.6±70.86	52.98±38.67	56.84±34.8	162.4 ± 65.74	150.8 ±69.6	27.46 ± 11.6	28.62 ± 15.47	7 points
8	El Khouly et al., 2016 [25]	Eygpt	200	26	174	Cohort	219.26± 26.76	198.2± 21.09	182.46±16.62	139.04±14.09	49.65±3.4	55.21 ± 10.98	192.65 ± 21.49	177.28 ± 29.45	36.49±3.32	27.81±2.82	8 points
9	Enaruna et al., 2014 [26]	Nigeria	120	80	40	Case- control	151.9± 7.45	140.14 ± 9.43	106.44±20.86	92.95±8.81	54.55±5.44	62.23±8.58	77.85±8.82	55.91±7.21	21.29±4.17	18.59 ±1.76	6 points
10	Irinyenikan et al., 2014 [27]	Nigeria	100	50	50	Case- control	188.54± 41.95	185.52 ± 38.19	196.61±78.55	151.06±47.5	32.85±12.14	28.10±9.66	116.13±35.06	127.08±32.88	39.32±15.71	31.00 ± 9.56	7 points
11	Nyam et al., 2014 [28]	Nigeria	100	50	50	Cross-sectional	175.56± 37.9	177.88 ± 40.22	172.72±78.83	90.35±45.17	30.55±11.6	67.29± 17.4	71.54 ± 14.7	73.47 ± 15.08	34.54 ±15.77	18.07 ± 9.03	6 points
12	Ephraim et al., 2014 [29]	Ghana	110	60	50	Case- control	293.9± 85.07	116 ± 69.6	451.7±141.72	194.86±141.7	92.8±73.47	100.54 ±65.74	139.2 ± 73.47	81.2 ± 54.14	93.2 ± 38.67	38.97 ± 28.34	6 points
13	Onuegbu et al., 2014 [30]	Nigeria	70	35	35	Case-control	216.16±35.58	179.04±30.16	251.55±39.86	165.63±33.66	42.54±4.64	48.72±7.80	61.1±31.32	56.07±30.16	50.31±7.97	33.13±6.73	5 points
14	Abubakar, 2011 [31]	Nigeria	100	50	50	Case-control	157.77± 19.14	137.28 ± 24.61	372.9 ± 78.83	73.52 ± 60.23	38.67 ± 12.76	130.7 ± 8.12	116.78 ± 26.68	105.18 ± 13.15	74.58 ±15.77	14.7 ± 12.05	5 points
15	Vanderjagt et al., 2004 [15]	Nigeria	173	43	130	Case- control	182.9± 50.71	190.64 ± 52.90	202.83±69.69	190.43±70.60	54.91±17.75	63.42 ± 17.64	145 ± 48.17	152.75 ± 48.50	40.57 ±13.94	38.09±14.14	5 points

HDL-High density lipoprotein, LDL-Low density lipoprotein, mg/dL-Milligram per deciliter, n-sample size, NP-Normal pregnant, PE-Pre-eclampsia, TG-Triglyceride, TC-Total cholesterol, VLDL- Very low density lipoprotein and data are expressed in mean ± Standard deviation.

### 3.3. Association of different variables with pre-eclampsia

In this analysis, we compared the mean values of variables among preeclamptic and normal pregnant women. Statistical significant difference was not observed between the two groups in regards to the mean values of maternal age, and gestational age. However, statistical significant difference were observed in the mean values of systolic blood pressure (SBP), diastolic blood pressure (DBP), TC, TG, LDL-C and VLDL-C levels between preeclamptic and normotensive pregnant women (Table 2).

**Table 2:** Paired sample test analysis of variables in PE and normotensive pregnant women.

S. No	Variable	Studies	Cases (N = 828) (Mean ± SD)	Controls (N = 1273) (Mean ± SD)	P-value
1	Age in year	15	28.69 ± 3.08	28.07 ± 4.49	0.181
2	GA in week	14	30.20 ± 9.22	29.54 ± 8.82	0.226
3	BMI	14	27.20 ± 3.45	25.61 ± 2.02	0.041**
4	SBP	7	164.53 ± 5.31	116.22 ± 2.90	0.000**
5	DBP	7	105.81 ± 3.82	71.61 ± 4.35	0.000**
6	TC (mg/dL)	15	221.46 ± 45.90	189.67 ± 39.18	0.024**
7	TG (mg/dL)	15	229.61 ± 88.27	147.00 ± 40.47	0.002**
8	HDL-c (mg/dL)	15	51.02 ± 16.01	61.80 ± 25.63	0.114
9	LDL-c (mg/dL)	15	133.92 ± 38.77	112.41 ± 36.08	0.003**
10	VLDL-c (mg/dL)	15	41.44 ± 19.68	26.64 ± 7.87	0.007**

BMI-Body mass index, DBP-Diastolic blood pressure, GA-Gestational age, HDL-c- High density lipoprotein-cholesterol, LDL-c- Low density lipoprotein-cholesterol, SD-Standard deviation, SBP-Systolic blood pressure, TC-Total cholesterol, TG-Triglyceride, VLDL-c- Very low density lipoprotein-cholesterol and ** Statistically significant at p<0.05.

### 3.4. Association of serum total cholesterol with pre-eclampsia

**Fig 2:**
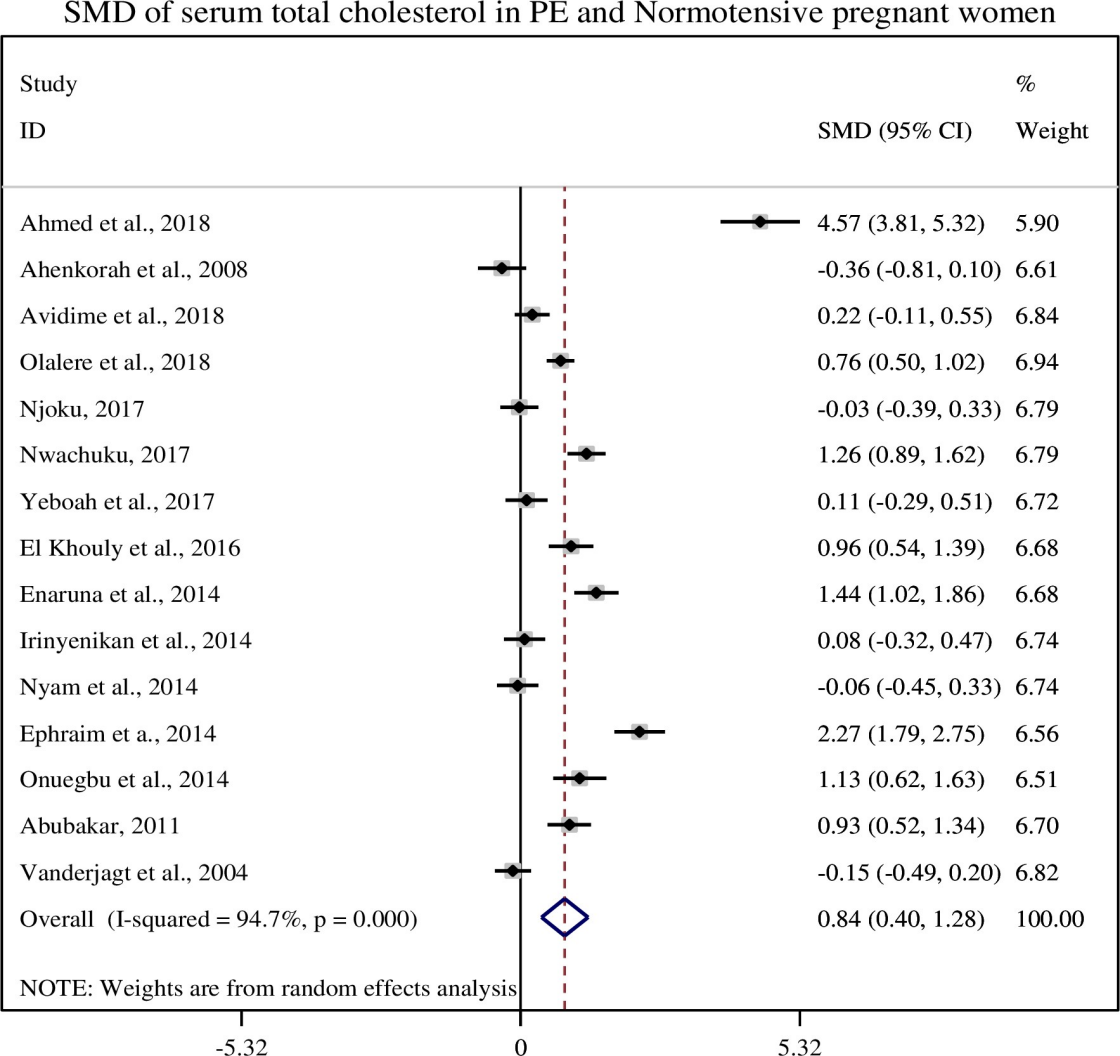
Forest plot of standardized mean difference of total cholesterol and pre-eclampsia.

In this sub-categorical analysis 15 studies were included to compare the serum levels of total cholesterol between pre-eclampsia and normotensive pregnant women [12, 14, 15, 20–31]. Eight of the included studies [12, 22, 24–26, 29–31] reported significantly higher serum levels of total cholesterol in pre-eclamptic women. Although, seven studies were showed non-significant association between the serum levels of total cholesterol and pre-eclampsia [14, 15, 20, 21, 23, 27, 28]. The pooled meta-regression analysis showed that there is a statistical significant association between total cholesterol and pre-eclampsia as compared with normotensive pregnant women with the pooled SMD of 0.84 (95% CI: 0.40, 1.29) (Fig 2).

### 3.5. Association of serum triglycerides with pre-eclampsia

**Fig 3:**
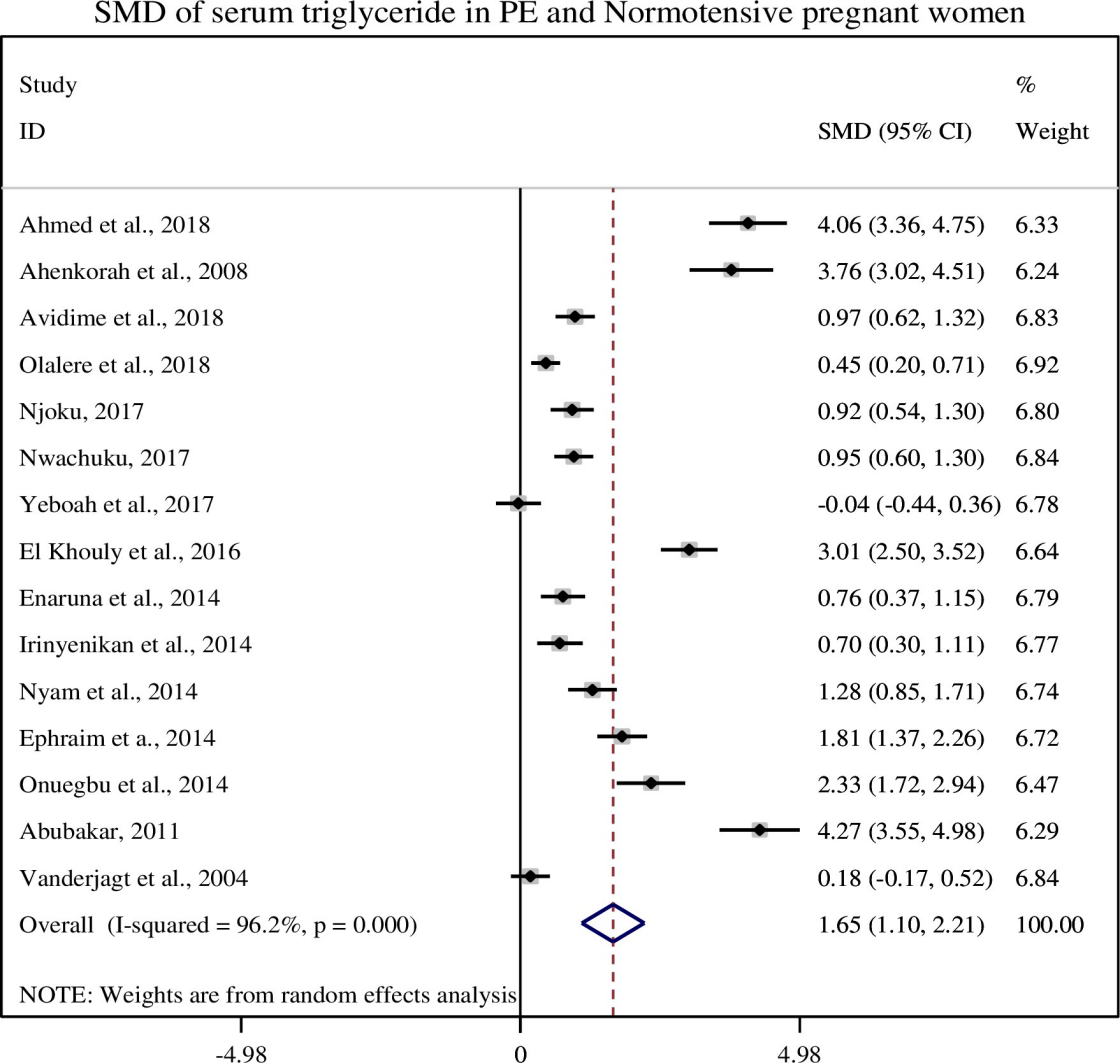
Forest plot of standardized mean difference of total triglycerides and pre-eclampsia.

In this sub-categorical analysis 15 studies were included to compare the serum triglyceride levels between pre-eclamptic and normotensive pregnant women [12, 14, 15, 20–31]. Thirteen of the included studies [12, 20–31] showed significantly higher serum triglyceride level in pre-eclamptic group as compared with normotensive pregnant women but two studies did not show significant association between serum triglyceride level and pre-eclampsia [14, 15]. The pooled meta-regression analysis showed that there is a statistical significant association between serum levels of triglycerides and pre-eclampsia as compared to normotensive pregnant women with the pooled SMD of 1.65 (95% CI: 1.10, 2.21) (Fig 3).

### 3.6. Association of serum HDL-cholesterol with pre-eclampsia

**Fig 4:**
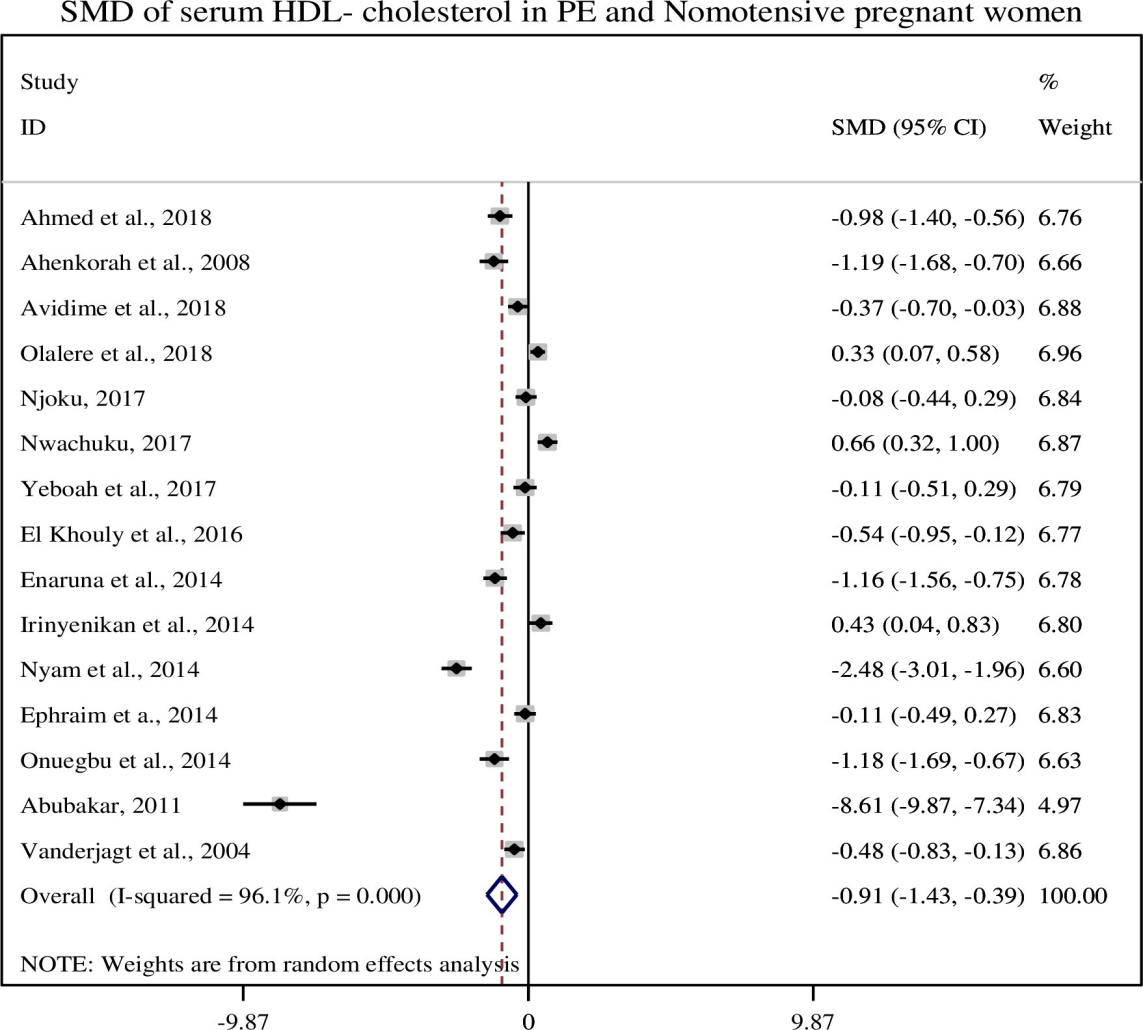
Forest plot of standardized mean difference of HDL-cholesterol and pre-eclampsia.

In this sub-categorical analysis 15 studies were included to compare the serum HDL-c levels between pre-eclamptic and normotensive pregnant women [12, 14, 15, 20–31]. Nine of the included studies [12, 15, 20, 21, 25, 26, 28, 30, 31] showed significantly lower levels of serum HDL-c and three studies [22, 24, 27] showed higher levels of serum HDL-c in pre-eclamptic group as compared to normotensive pregnant women but, three studies [14, 23, 29] did not show a significant association between serum HDL-c and pre-eclampsia. The pooled meta-regression analysis showed that there is a statistical significant association between serum levels of HDL-c and pre-eclampsia as compared to normotensive pregnant women with the pooled SMD of -0.91 (95% CI: -1.43, -0.39) (Fig 4).

### 3.7. Association of serum LDL-cholesterol with pre-eclampsia

**Fig 5:**
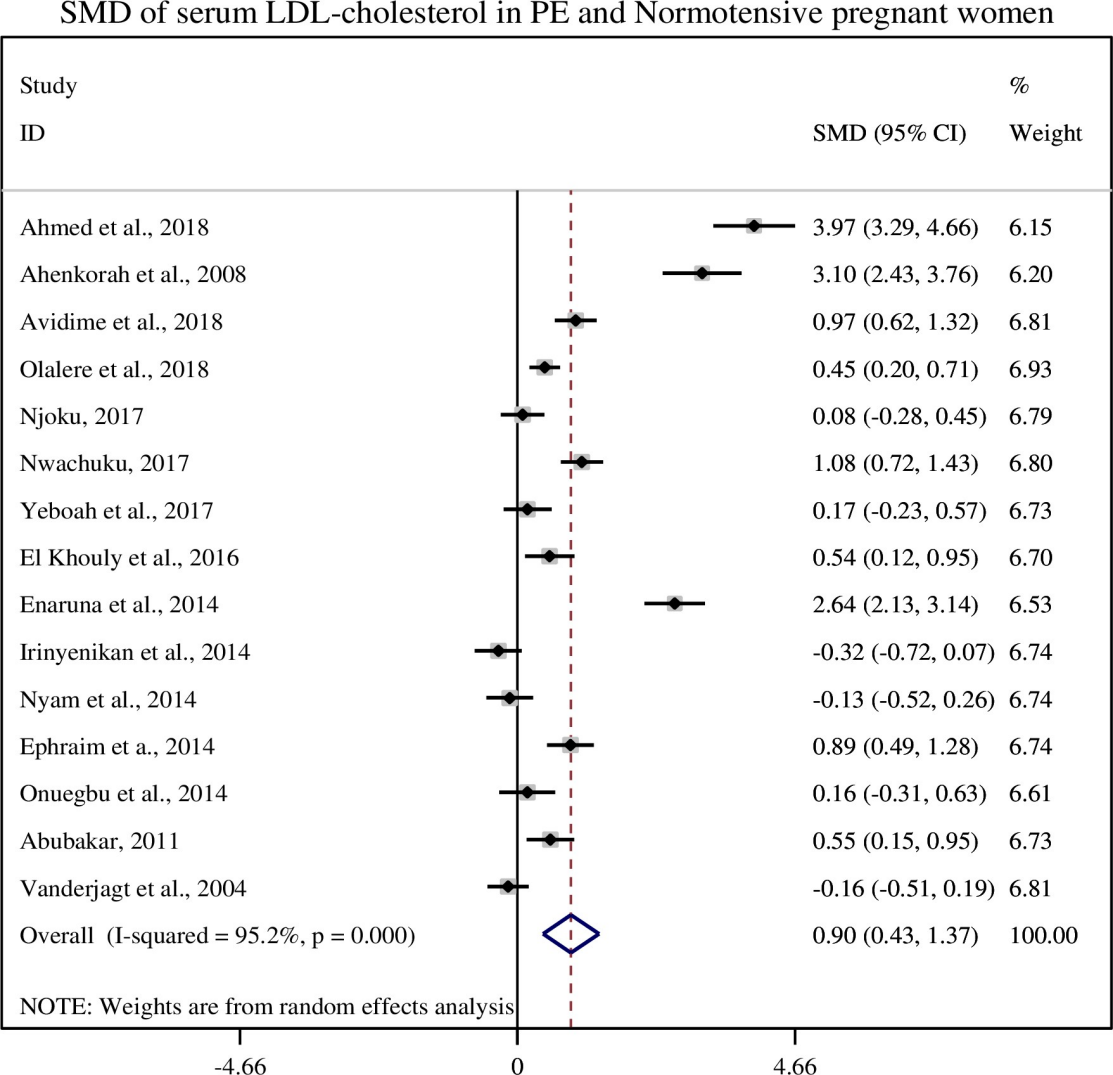
Forest plot of standardized mean difference of LDL-cholesterol and pre-eclampsia.

In this sub-categorical analysis 15 studies were included to compare the serum LDL-cholesterol levels between pre-eclamptic and normotensive pregnant women [12, 14, 15, 20–31]. Nine of the included studies [12, 20–22, 24–26, 29, 31] showed significantly higher serum levels of LDL-c in pre-eclamptic group, but six studies didn’t show significant association between serum levels of LDL-c and pre-eclampsia [14, 15, 23, 27, 28, 30]. The pooled meta-regression analysis showed that there is a statistical significant association between the serum levels of LDL-c and pre-eclampsia as compared to normotensive pregnant women with the pooled SMD of 0.95 (95% CI: 0.46, 1.45) (Fig 5).

### 3.8. Association of serum VLDL-cholesterol with pre-eclampsia

**Fig 6:**
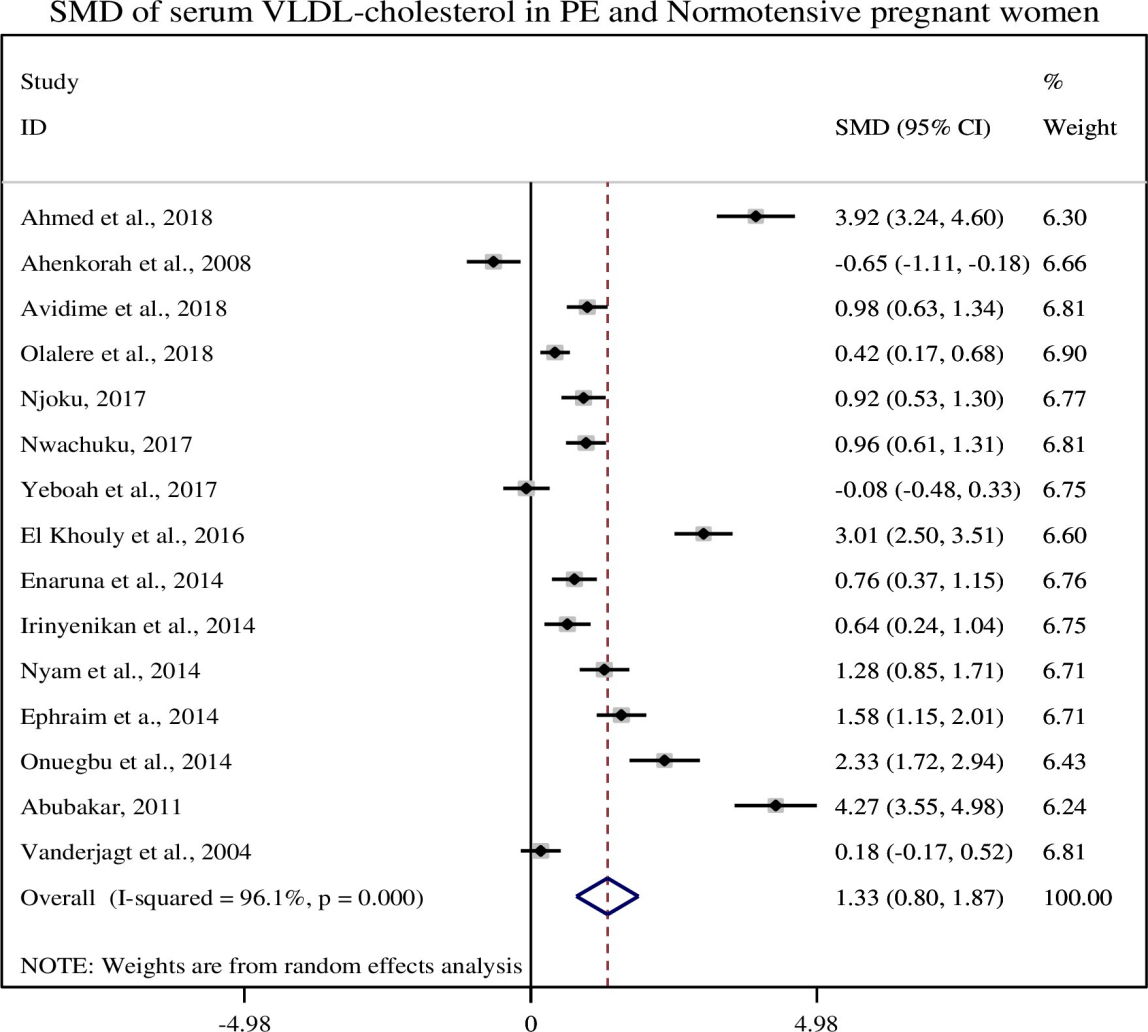
Forest plot of standardized mean difference of VLDL-cholesterol and pre-eclampsia.

In this sub-categorical analysis 15 studies were included to compare the serum level of VLDL-c between pre-eclampsia and normotensive pregnant women [12, 14, 15, 20–31]. Twelve studies [12, 21–31] showed significantly higher serum levels of VLDL-c in pre-eclampsia whereas one study [20] showed lower level of serum VLDL-c in pre-eclampsia. Although, two studies [14, 15] did not show significant association between the serum level of VLDL- c and pre-eclampsia. Pooled meta-regression analysis showed that there is a statistical significant association between the serum levels of VLDL-c and pre-eclampsia as compared to normotensive pregnant women with a pooled SMD of 1.27 (95% CI: 0.72, 1.81) (Fig 6).

### 3.9. Sensitivity analysis and publication bias

A sensitivity test was done by omitting one study at a time to assess the stability of the results. There was no significant change in the pooled SMD after excluding one of the studies at 95% CI. This means there is no individual study that excessively influences the pooled effects of the serum lipid profiles in pre-eclamptic women (S2 File). Due to the presence of high statistical heterogeneity random effect model was used for analysis. To sort out the cause heterogeneity, publication bias was assessed by funnel plot and Egger’s test. Funnel plot and Egger’s test didn’t show evidence of publication bias between the serum levels of lipid profile and the risks of pre-eclampsia (S3 File). However, the Egger’s test of LDL-cholesterol shows the presence of possible publication bias (p = 0.010). This bias might be due to missing of grey literatures and exclusion of non-English language articles.

## 4. Discussion

This study is the first systematic review and meta-analysis in African pregnant women which provides information on the association of maternal serum lipid profiles with pre-eclampsia. In this study, maternal age and gestational age were comparable between the two groups and these variables didn’t show a statistical significant association with pre-eclampsia. The mean BMI, systolic and diastolic blood pressure measurements were significantly different among pre-eclamptic and normotensive pregnant women.

The cause of pre-eclampsia is not well understood but it is believed that reduced uteroplacental perfusion as a result of abnormal spiral artery remodeling causes the disease. Placental ischemia causes an increased synthesis of endothelin, thromboxane and different chemical mediators which affects the endothelium and resulted maternal syndromes [32]. It is a known fact that lipid metabolism is markedly changed during pregnancy and dyslipidemia involved in the pathogenesis of pre-eclampsia [33]. There are different serum lipid biomarkers that predict the risk of pre-eclampsia in pregnant women [34]. In the early pregnancy, the mother stayed in anabolic state but in the late pregnancy the mother is in the catabolic state which leads to an increased in the concentration of plasma free fatty acids and glucose level due to insulin resistance [6]. The plasma lipid levels are increased due to hormonal changes and the problem worsens as the gestational age increases [35].

In this review, we found that the mean serum total cholesterol level was significantly higher in preeclamptic women as compared to normotensive pregnant women (221.84 ± 47.61 and 190.43 ± 40.55), respectively and its pooled SMD was (SMD = 0.82, 95% CI: 0.35, 1.29). Similar results were reported in the studies conducted in China and India [36, 37]. The plasma cholesterol level increases during pregnancy in response to an increase estrogen induced hepatic synthesis or failure of lipoprotein lipase to clear the plasma lipids. Higher plasma cholesterol level is used for placental steroid, placental membrane synthesis and stored as maternal fat store which served as a fuel for the mother as well as for the growing fetus in later pregnancy or during lactation [35, 36].

The mean serum triglyceride level of the current study was significantly higher in preeclamptic women than normotensive pregnant women (228.04 ± 91.38 and 145.67 ± 121.61), respectively and its pooled SMD was (SMD = 1.61, 95% CI: 1.03, 2.14). Similar, findings were reported in the studies conducted in China and Pakistan [36, 38, 39]. In the prospective cohort study conducted in Netherlands, hypertriglyceridemia were associated with pre-eclampsia and high blood pressure in the early pregnancy [40]. During the course of pregnancy the levels of triglyceride is also increases in response to estrogen and hepatic lipase activity. Additionally, a reduced lipoprotein lipase enzyme activity and insulin resistance leads to decrease in lipid catabolism at the tissue level which causes hypertriglyceridemia [41, 42]. Hypertriglyceridemia is an important risk factor for cardiovascular disease (CVD), hypertension, diabetes and metabolic syndrome in obese and insulin resistant persons [43].

In this review, we found that the mean serum HDL-c level was lower in preeclamptic women as compared to normotensive pregnant women (51.62 ± 16.44 and 62.74 ± 26.33), respectively and its pooled SMD was (SMD = -0.89, 95% CI: -1.43, -0.35). Similar findings were reported in the studies conducted in Nigeria and China [21, 36]. Reports showed that reduced levels of HDL-c being associated with an increased risk of coronary disease and myocardial infarction [44]. HDL carries cholesterol from peripheral tissues to the liver, where it is broken down for excretion and used for synthesis of biomolecules. Higher levels of HDL lipoprotein have protective effect against hypertension and cardiovascular diseases [45].

In the current study, we found that the mean serum LDL-cholesterol level was significantly higher in preeclamptic women than normotensive pregnant women (139.12 ± 34.38 and 116.43 ± 33.77), respectively and its pooled SMD was (SMD = 0.95, 95% CI: 0.46, 1.45). Similar, evidences were reported in the studies conducted in China, Bangladesh and Nigeria [21, 36, 46]. LDL-c transports cholesterol to the peripheral tissue and plays significant role in the development of atherosclerosis and cardiovascular disease [47]. The oxidized LDL products modify the lysine residues of Apo-lipoprotein B (apo B) which is recognized by the receptors of macrophages. The modified product of LDL is engulfed by macrophages and converted into foam cells which induce production of different inflammatory mediators resulted in plaque formation and atherosclerosis [48].

In this review, we found that the mean serum VLDL-cholesterol level was significantly higher in preeclamptic women than normotensive pregnant women (40.81 ± 20.26 and 26.18 ± 7.96), respectively and its pooled SMD was (SMD = 1.27, 95% CI: (0.72, 1.81)). Similar finding were reported in the studies conducted in China and Nigeria [21, 36]. VLDL cholesterol carries the highest amount of triglycerides from the liver to the blood vessels which have been linked to atherosclerosis and the subsequent risk of heart diseases and stroke [45]. VLDL cholesterol remnants are associated with the risk of pre-eclampsia and high blood pressure [40]. VLDL and LDL cholesterol are known as bad lipoprotein to due to an increased risk of atherosclerosis. Experts suggested that lower levels of VLDL and LDL cholesterol and higher levels of HDL cholesterol are beneficial for the prevention of chronic diseases.

## 5. Strength and limitation

### Strength

This systematic review and meta-analysis generated pooled data showing lipid profiles and risk of pre-eclampsia in African women. In addition, this review serves as baseline information for further study.

### Limitation

The search strategy was limited to articles published in English, and this could lead to reporting bias. We include small number of studies and most of them were from Nigeria, and this may influence its generalizability to African women. Moreover, presence of high statistical heterogeneity among the included studies would decrease the generalization of evidence from this review.

## 6. Conclusion

In this systematic review and meta-analysis the mean serum lipid profiles were significantly higher in pre-eclamptic women than in normotensive pregnant women. The pooled standardized mean difference of serum lipid profiles such as: total cholesterol, triglyceride, LDL-cholesterol, and VLDL-Cholesterol were significantly higher in pre-eclamptic women than normotensive pregnant women but HDL-cholesterol was lower in preeclamptic women. Thus, we conclude that dyslipidemia could play certain roles in the pathogenesis of pre-eclampsia. However, concrete evidence on the roles of dyslipidemia in pre-eclampsia in African pregnant women should require large scale prospective studies.

## Supporting information

S1 Checklist PRISMA check list of the review. (DOC)

S1 Table Quality assessment. (XLSX)

S1 File Subgroup analysis of the review. (DOCX)

S2 File Sensitivity test of the review. (DOCX)

S3 File Funnel plot of lipid profiles. (DOCX)

S4 File Search strategy. (DOCX)

## Abbreviations

BMI: body mass index
CI: confidence interval
DBP: diastolic blood pressure
GA: gestational age
HDL: high density lipoprotein
JBI: MAStARI, Joanna Briggs Institute Meta-Analysis of Statistics Assessment and Review Instrument
LDL: low density lipoprotein
NOS: Newcastle-Ottawa Scale
PE: Pre-eclampsia
PRISMA: preferred reporting items for systematic reviews and meta-analyses
RR: risk ratio
SBP: systolic blood pressure
SD: Standard Deviation
SEM: standard error of Mean
SMD: standardized mean difference
TC: total cholesterol
TG: triglycerides and VLDL, very low density lipoprotein
