# Supplementary material for: Maternal lipid profile and risk of pre-eclampsia in African pregnant women: A systematic review and meta-analysis
Source: PLoS One. 2020 Dec 23;15(12):e0243538. doi: 10.1371/journal.pone.0243538 (PMC7757810; doi:10.1371/journal.pone.0243538)
Supplement: S1 Checklist — (DOC) [file pone.0243538.s001.doc]

| **Section/topic** | **#** | **Checklist item** | **Reported on page #** |
| --- | --- | --- | --- |
| **TITLE** | | |  |
| Title | 1 | **Maternal Lipid Profile and Risk of Pre-eclampsia in African Pregnant Women: A systematic Review and Meta- analysis.** | 1 |
| **ABSTRACT** | | |  |
| Structured summary | 2 | **Introduction:** Some studies have reported the association between maternal serum lipid profile abnormalities and pre-eclampsia. However, many studies have reported controversial results. Hence, this systematic review and meta-analysis was planned to generate summarized evidence on the association between maternal serum lipid profiles and pre-eclampsia in African women.  **Methods:** Four electronic databases such as; PubMed, Hinari, Google Scholar, and African Journals Online were searched for studies published in English. Joanna Briggs Institute Meta-Analysis of Statistics Assessment and Review Instrument and Newcastle-Ottawa Scale were used for data extraction and quality assessment of the included studies. The meta- regression analysis was performed by Stata 14 software. Standardized mean difference (SMD) values of lipid profiles were computed to assess their association with pre-eclampsia at 95% CI.  **Results:** In this review a total of 15 observational studies were included. The mean levels of triglyceride (TG), total cholesterol (TC), low density lipoprotein- cholesterol (LDL-c) and very low density lipoprotein- cholesterol (VLDL-c) were significantly higher in pre-eclamptic women as compared with normotensive pregnant women (TG= 229.61±88.27 and 147.00 ± 40.47, TC= 221.46 ± 45.90 and 189.67 ± 39.18, LDL= 133.92 ± 38.77 and 112.41 ± 36.08, VLDL= 41.44 ± 19.68 and 26.64 ± 7.87), respectively. On the other hand, the maternal serum high density lipoprotein cholesterol (HDL-c) level was lower, but it is not statistically significant (HDL-c 51.02 ± 16.01 and 61.80 ± 25.63) in pre-eclamptic women as compared with controls. The pooled standardized mean difference (SMD) of TG, TC, LDL-C and VLDL-C were significantly increased in pre-eclamptic women as compared with normotensive pregnant women with the SMD of (TG =1.65 (1.10, 2.21), TC= 0.84 (0.40, 1.29), LDL-C = 0.95 (0.46, 1.45) and VLDL-C = 1.27 (0.72, 1.81)) at 95% CI, respectively. Instead the pooled SMD of HDL-c was small in pre-eclamptic women (SMD= -0.91 (95% CI: -1.43, -0.39).  **Conclusion:** In this review, maternal serum levels of TG, TC, LDL-c and VLDL-c were significantly associated with the risk of preeclampsia. However, HDL-c was not significantly associated and it was low in pre-eclamptic African women. Future large scale prospective studies should verify these outcomes and it is recommended that lipid profiles should be included as a routine diagnostic test for pre-eclampsia.  **Key terms:** Lipid profiles, Pre-eclampsia, Meta-analysis, Africa | 2 |
| **INTRODUCTION** | | |  |
| Rationale | 3 | Pre-eclampsia is a pregnancy related metabolic syndrome characterized by hypertension and proteinuria occurred after 20 weeks of gestation. It is the most common cause of maternal and prenatal morbidity and mortality (1). The causes of pre-eclampsia is not clearly known, but failure of spiral artery remodeling leads to placental ischemia which causes maternal syndromes hypertension and proteinuria (2). Increased in lipid oxidation products and decreased in the levels of antioxidants involved in the pathogenesis of pre-eclampsia (3). Many evidences showed that the risk of pre-eclampsia increases in the women with higher levels of oxidized low-density lipoprotein (LDL) and triglycerides (TG) and lower levels of circulating vitamin C as compared to normotensive pregnant women (4, 5). The oxidative conversion of LDL- cholesterol to oxidized LDL form is considered to be a key event for the initiation and development of atherosclerosis and hypertension (4).  Early pregnancy dyslipidemia is associated with an increased risk of pre-eclampsia (1). In the early pregnancy, the mother exists in the anabolic state and the lipid serves as a source of calories for the growing fetus as well as for the mother in the third trimester (6). There are many facts that show lipid profile abnormalities might be associated with the risk of pre-eclampsia. Lipid profiles such as: TG, LDL-c, total cholesterol (TC) and very low density lipoprotein (VLDL) levels are higher in pre-eclamptic women as compared with normal pregnancies, but high-density lipoprotein (HDL) level is lower in pre-eclamptic women as compared with normal pregnancies (7-9). Lipid profile abnormalities increases as the gestational age of the mother increases. A meta-analysis study which consisted of 24 case control studies confirmed a strong association between hypertriglyceridemia and the risk of pre-eclampsia (10).  Different studies have tried to see any associations between the maternal serum lipid profiles and pre-eclampsia, but many of them have reported conflicting results. Some studies have shown significant higher levels of lipid profiles in pre-eclamptic women as compared to normotensive pregnant women (9, 11-13). However, a few studies have found non-significant difference of serum level of maternal lipid profiles between pre-eclamptic and normotensive pregnant women (14, 15) and few other reports indicated lower lipid profiles in pre-eclamptic women as compared to normotensive controls. | 3-4 |
| Objectives | 4 | This systematic review and meta-analysis was designed to generate summarized evidence on the association of serum maternal lipid profiles and the risk of pre-eclampsia in African pregnant women. | 4 |
| **METHODS** | | |  |
| Protocol and registration | 5 | This review protocol is registered at the National Institute for Health Research; PROSPERO international prospective register of systematic reviews with registration number CRD42020192865 at ([https://www.crd.york.ac.uk/prospero/#recordDetails](https://www.crd.york.ac.uk/prospero/#recordDetails)). | 4 |
| Eligibility criteria | 6 | 1. Studies conducted on human subjects were included. 2. Studies with case-control, cross-sectional and cohort designs in Africa were included. 3. Articles that report pre-eclampsia as an outcome variable were included. 4. Published articles written in English were included. 5. Studies considering lipid profiles as the determinant variable and reporting the result in mean and standard deviation were included. 6. Studies conducted in the community or in the health institution were included. 7. Conference papers, editorials, reviews and randomized control trials were excluded. | 4 |
| Information sources | 7 | We searched the following databases: PubMed, Hinari, Google Scholar, and African Journals Online (AJOL). All published studies up-to July 30, 2020 in Africa were retrieved to be assessed for eligibility of inclusion in this review. | 4 |
| Search | 8 | The search was done by using Medical Subject Heading (MeSH) terms*;* ““Lipids, Triglycerides, Cholesterol, HDL, VLDL, LDL, Pre-eclampsia and Africa*”* separately or in combination. | 4 |
| Study selection | 9 | All citations identified by our search strategy were exported to EndNote -X9- and duplicate articles were removed. And then the titles and abstracts of the identified articles were screened by two independent reviewers, and eligible studies were included for further review. The full texts of selected articles were retrieved and read thoroughly to ascertain the suitability prior to data extraction. In case of disagreement between the two reviewers, discussion was held to reach consensus and the third reviewer was consulted. The search process was presented in PRISMA flow chart that clearly shows the studies that were included and excluded with reasons of exclusion (Fig. 1) (16). | 5 |
| Data collection process | 10 | Data from the selected articles were extracted by two independent reviewers by using excel data extraction sheet. Any discrepancy between the two reviewers was solved by discussion. | 6 |
| Data items | 11 | Data extraction includes: author’s name, publication year, study country, study design, sample size, number of cases and controls, mean age, mean gestational age, mean BMI, mean SBP and DBP, mean TG, TC, HDL, LDL and VLDL levels were extracted**.** | 6 |
| Risk of bias in individual studies | 12 | To assess risk of bias, two authors independently used the modified Newcastle Ottawa Scale for risk of bias assessment tool. Each item scored one point and discrepancies were resolved by discussion. | 6 |
| Summary measures | 13 | Transformed standardized mean difference with 95% CI was the summary measure used. | 7 |
| Synthesis of results | 14 | The analysis was done through using Stata 14 statistical software. Random effect model analysis was used for analysis. We used the Standardized mean difference of the serum lipid profiles to compute the association serum lipid profiles with pre-eclampsia in African pregnant women. | 7 |

Page 1 of 2

| **Section/topic** | **#** | **Checklist item** | **Reported on page #** |
| --- | --- | --- | --- |
| Risk of bias across studies | 15 | Statistical heterogeneity was estimated through Cochrane Q, I^2^ statistic and P-value. If I^2^ statistic value < 25%, 25-50%, and ≥50% was used to declare the heterogeneity test as low, medium and high heterogeneity. Risk of bias across the studies was assessed by using funnel plot and Egger’s test. | 7 |
| Additional analyses | 16 | To know the presence of heterogeneity sub-group and sensitivity test were performed. | 7 |
| **RESULTS** | | |  |
| Study selection | 17 | A total of 537 articles were retrieved through electronic search by using different search terms of which 454 article were eligible for title and abstract assessment after removal of 83 duplicate records. Out of 454 articles screened for eligibility 414 records were excluded after assessing their title and abstract. A total of 40 articles underwent full- text assessment, 25 studies were excluded due to different reasons (21 articles were done outside the study area and four articles didn’t report outcome variable). | 7 |
| Study characteristics | 18 | In this review a total of 15 studies were included. Ten of them were case-control and three studies were Cross-sectional and the other two were cohort. Studies that have been conducted in Africa and published up- to July 30, 2020 were included. Ten studies were conducted in Nigeria and the other three and two studies were conducted in Ghana and Egypt respectively. In this review a total of 2,106 pregnant women were included (828 cases and 1, 273 controls) (**Table 1**). | 7 |
| Risk of bias within studies | 19 | Statistical heterogeneity was estimated through Cochrane Q, I^2^ statistic and P-value. If I^2^ statistic value < 25%, 25-50%, and ≥50% was used to declare the heterogeneity test as low, medium and high heterogeneity. In this review, random effect model (REM) was used for analysis. | 15 |
| Results of individual studies | 20 | **Association of serum total cholesterol with pre-eclampsia**  In this sub-categorical analysis 15 studies were included to compare the serum levels of total cholesterol between pre-eclampsia and normotensive pregnant women (12, 14, 15, 20-31). Eight of the included studies (12, 22, 24, 25, 28-31) reported significantly higher serum levels of total cholesterol in pre-eclamptic group. Although, seven studies were showed non-significant association between the serum levels of total cholesterol and pre-eclampsia (14, 15, 20, 21, 23, 26, 27). The pooled meta-regression analysis showed that there is a statistical significant association between total cholesterol and pre-eclampsia as compared to normotensive pregnant women with a pooled SMD of 0.84 (95% CI: 0.40, 1.29) **(Fig. 2).**  **Association of serum triglycerides with pre-eclampsia**  In this sub-categorical analysis 15 studies were included to compare the serum triglycerides level between pre-eclampsia and normotensive pregnant women (12, 14, 15, 20-31). Thirteen of the included studies (12, 20-31) showed significantly higher serum triglyceride level in pre-eclamptic group as compared to normotensive pregnant women but two studies did not show significant association between serum triglyceride level and pre-eclampsia (14, 15). Pooled meta-regression analysis showed that there is a statistical significant association between serum levels of triglycerides and pre-eclampsia as compared to normotensive pregnant women with a pooled SMD of 1.65 (95% CI: 1.10, 2.21) **(Fig. 3).**  **Association of serum HDL-cholesterol with pre-eclampsia**  In this sub-categorical analysis 15 studies were included to compare the serum HDL-c level between pre-eclampsia and normotensive pregnant women (12, 14, 15, 20-31). Nine of the included studies (12, 15, 20, 21, 25, 27, 29-31) showed significantly lower levels of serum HDL-c and three studies (22, 24, 26) showed higher levels of serum HDL-c in pre-eclamptic group as compared to normotensive pregnant women but, three studies (14, 23, 28) did not show a significant association between serum HDL-c and pre-eclampsia. Pooled meta-regression analysis showed that there is a statistical significant association between serum levels of HDL-c and pre-eclampsia as compared to normotensive pregnant women with a pooled SMD of -0.91 (95% CI: -1.43, -0.39) **(Fig. 4).**  **Association of serum LDL-cholesterol with pre-eclampsia**  In this sub-categorical analysis 15 studies were included to compare the serum LDL-cholesterol level between pre-eclampsia and normotensive pregnant women (12, 14, 15, 20-31). Nine of the included studies (12, 20-22, 24, 25, 28, 30, 31) showed significantly higher serum levels of LDL-c in pre-eclamptic group, but six studies did not show significant association between serum levels of LDL-c and pre-eclampsia (14, 15, 23, 26, 27, 29). Pooled meta-regression analysis showed that there is a statistical significant association between the serum levels of LDL-c and pre-eclampsia as compared to normotensive pregnant women with a pooled SMD of 0.95 (95% CI: 0.46, 1.45) **(Fig. 5).**  **Association of serum VLDL-cholesterol with pre-eclampsia**  In this sub-categorical analysis 15 studies were included to compare the serum level of VLDL-c between pre-eclampsia and normotensive pregnant women (12, 14, 15, 20-31). Twelve studies (12, 21-31) showed significantly higher serum levels of VLDL-c in pre-eclampsia whereas one study (20) showed lower level of serum VLDL-c in pre-eclampsia. Although, two studies (14, 15) did not show significant association between the serum level of VLDL- c and pre-eclampsia. Pooled meta-regression analysis showed that there is a statistical significant association between the serum levels of VLDL-c and pre-eclampsia as compared to normotensive pregnant women with a pooled SMD of 1.27 (95% CI: 0.72, 1.81) **(Fig. 6).** | 9-14 |
| Synthesis of results | 21 | In this review we had estimated the standardized mean difference of lipid profiles at 95% CI with pre-eclampsia in African pregnant women. | 7-12 |
| Risk of bias across studies | 22 | Egger’s test and funnel plot were used to assess the risk of bias across the studies | 15 |
| Additional analysis | 23 | None | 12-15 |
| **DISCUSSION** | | |  |
| Summary of evidence | 24 | This study is the first systematic review and meta-analysis in Africa providing information on the association of maternal serum lipid profiles with pre-eclamptic and normotensive pregnant women. In this study, maternal age and gestational age were comparable between the two groups and these variables were not statistically associated with pre-eclampsia. The mean BMI, systolic and diastolic blood pressure measurements in pre-eclamptic women were not significantly different from those of normotensive pregnant women.  The cause of pre-eclampsia is not clearly known but it has been understood that reduced uteroplacental perfusion is a result of abnormal spiral artery remodeling. Placental ischemia causes an increased synthesis of endothelin, thromboxane and different chemical mediators which affects the endothelium and resulted maternal syndromes (32). It is a well-known fact lipid metabolism is markedly changed during pregnancy and dyslipidemia involved in the pathogenesis of pre-eclampsia (33). There are different serum lipid biomarkers that predict the risk of pre-eclampsia in the pregnant women (34). In the early gestation, the mother is stayed in anabolic state but in the late pregnancy the mother is in the catabolic state which causes an increase in the concentration of plasma free fatty acid and glucose level due to insulin resistance (6). The plasma lipid levels is increased due to hormonal changes and the problem worsens if the gestational age increased (35).  In this review, we found that the mean serum total cholesterol level was significantly higher in preeclamptic women than in normotensive pregnant women (221.84 ± 47.61 and 190.43 ± 40.55), respectively and its pooled SMD was (SMD= 0.82, 95% CI: 0.35, 1.29). Similar results were reported in the studies that were conducted in China and India (36, 37). The plasma cholesterol level increases during pregnancy in response to an increase estrogen induced hepatic synthesis or failure of lipoprotein lipase to clear the plasma lipids. Increased plasma cholesterol level is used for placental steroid synthesis, placental membrane synthesis and stored as maternal fat store which serves as a fuel for the mother as well as for the growing fetus in later pregnancy or during lactation (35, 36).  The mean serum triglyceride level of the current study was significantly higher in preeclamptic women than in normotensive pregnant women (228.04 ± 91.38 and 145.67 ± 121.61), respectively and its pooled SMD was (SMD= 1.61, 95% CI: 1.03, 2.14). Similar, findings were reported in the studies conducted in China and Pakistan (36, 38, 39). In the prospective cohort study conducted in the Netherlands pregnant women, hypertriglyceridemia were associated with pre-eclampsia and high blood pressure in the early pregnancy (40). During the course of pregnancy the levels of triglyceride is also increases in response to estrogen and hepatic lipase activity. Additionally, a reduced lipoprotein lipase enzyme activity and insulin resistance leads to decrease in lipid catabolism at the tissue level which causes hypertriglyceridemia (41, 42). Hypertriglyceridemia is an important risk factor for cardiovascular disease (CVD), hypertension, diabetes and metabolic syndrome in the obese and insulin resistance persons (43).  In this review, we found that the mean serum HDL-c level was lower in preeclamptic women compared than in normotensive pregnant women (51.62 ± 16.44 and 62.74 ± 26.33), respectively and its pooled SMD was (SMD= -0.89, 95% CI: -1.43, -0.35). Similar findings were reported in the studies conducted in Nigeria and China (21, 36). Reports show that reduced levels of HDL-c being associated with an increased risk of coronary disease and myocardial infarction (44). HDL lipoprotein carries cholesterol from peripheral tissues to the liver, where it is either be broken down or prepared to be excreted from the body as a waste product. High concentrations of HDL lipoprotein have protective function against hypertension and cardiovascular diseases (45).  In the current study, we found that the mean serum LDL-cholesterol level was significantly higher in preeclamptic women than in normotensive pregnant women (139.12 ± 34.38 and 116.43 ± 33.77), respectively and its pooled SMD was (SMD= 0.95, 95% CI: 0.46, 1.45). Comparable, level of evidences were reported in the studies conducted in China, Bangladesh and Nigeria (21, 36, 46). LDL-c transports cholesterol to the peripheral tissue and plays significant role in the development of atherosclerosis and cardiovascular disease (47). The oxidized LDL products modify the lysine residues of Apo-lipoprotein B (apo B) which is recognized by the scavenger receptors of the macrophages. The modified product of LDL is engulfed by macrophages and converted into foam cells. This foam cells produced different inflammatory mediators leading to plaque formation and atherosclerosis (48).  In this review, we found that the mean serum VLDL-cholesterol level was significantly higher in preeclamptic women than in normotensive pregnant women (40.81 ± 20.26 and 26.18 ± 7.96), respectively and its pooled SMD was (SMD= 1.27, 95% CI: (0.72, 1.81)). Similar levels of evidences were reported in the studies conducted in China and Nigeria (21, 36). VLDL contains the highest amount of triglycerides which have been linked to atherosclerosis and the subsequent risk of heart diseases and stroke (45). VLDL cholesterol remnant is associated with pre-eclampsia and high blood pressure (40). | 15-17 |
| Limitations | 25 | **Strength:** This systematic review and meta-analysis generated pooled data showing lipid profiles and risk of pre-eclampsia in African women. In addition, this review serves as baseline information for further study.  **Limitation:** The search strategy was limited to articles published in English, and this could lead to reporting bias. We include small number of studies and most of them were from Nigeria, and this may influence its generalizability to African women. Moreover, presence of high statistical heterogeneity among the included studies would decrease the generalization of evidence from this review. | 17 |
| Conclusions | 26 | In this systematic review and meta-analysis the mean serum lipid profiles were significantly higher in pre-eclamptic women than in normotensive pregnant women. The pooled standardized mean difference of serum lipid profiles such as: total cholesterol, triglyceride, LDL-cholesterol, and VLDL-Cholesterol were significantly higher in pre-eclamptic women than in normal pregnant women but HDL-cholesterol was lower in preeclamptic group. Thus, we deduced that dyslipidemia could play certain roles in the pathogenesis of pre-eclampsia. However, concrete evidences on the roles of dyslipidemia in pre-eclampsia in African pregnant women should require large scale prospective studies. | 17-18 |
| **FUNDING** | | |  |
| Funding | 27 | We did not receive any fund for this study. | 18 |
