# Supplementary material for: Maternal lipid profile and risk of pre-eclampsia in African pregnant women: A systematic review and meta-analysis
Source: PLoS One. 2020 Dec 23;15(12):e0243538. doi: 10.1371/journal.pone.0243538 (PMC7757810; doi:10.1371/journal.pone.0243538)
Supplement: S1 File — (DOCX) [file pone.0243538.s003.docx]

**Subgroup analysis of lipid profiles in PE and Normotensive pregnant women in Africa**

**Subgroup analysis of Total cholesterol in PE and Normotensive pregnant women**

**Fig.1** Forest plot of subgroup analysis of total cholesterol and pre-eclampsia.

**Subgroup analysis of triglycerides in PE and Normotensive pregnant women**

**Fig.2** Forest plot of subgroup analysis of total triglycerides and pre-eclampsia.

**Subgroup analysis of HDL-Cholesterol in PE and Normotensive pregnant women**

**Fig.3** Forest plot of subgroup analysis of HDL-Cholesterol and pre-eclampsia.

**Subgroup analysis of LDL-Cholesterol in PE and Normotensive pregnant women**

**Fig.4** Forest plot of subgroup analysis of LDL-Cholesterol and pre-eclampsia

**Subgroup analysis of VLDL-Cholesterol in PE and Normotensive pregnant women**

**Fig.5** Forest plot of subgroup analysis of VLDL-Cholesterol and pre-eclampsia
