# Supplementary material for: Maternal lipid profile and risk of pre-eclampsia in African pregnant women: A systematic review and meta-analysis
Source: PLoS One. 2020 Dec 23;15(12):e0243538. doi: 10.1371/journal.pone.0243538 (PMC7757810; doi:10.1371/journal.pone.0243538)
Supplement: S2 File — (DOCX) [file pone.0243538.s004.docx]

**Sensitivity test of serum level of lipid profiles in pre-eclamptic and normotensive pregnant women**

Fig.1 Sensitivity test for total cholesterol in pre-eclampsia and normotensive pregnant women

Fig. 2 Sensitivity test for triglycerides in pre-eclampsia and normotensive pregnant women

Fig.3 Sensitivity test for HDL-cholesterol in pre-eclampsia and normotensive pregnant women

Fig.4 Sensitivity test for LDL-cholesterol in pre-eclampsia and normotensive pregnant women

Fig.5 Sensitivity test for VLDL-cholesterol in pre-eclampsia and normotensive pregnant women
