# Supplementary material for: Maternal lipid profile and risk of pre-eclampsia in African pregnant women: A systematic review and meta-analysis
Source: PLoS One. 2020 Dec 23;15(12):e0243538. doi: 10.1371/journal.pone.0243538 (PMC7757810; doi:10.1371/journal.pone.0243538)
Supplement: S3 File — (DOCX) [file pone.0243538.s005.docx]

**Funnel plot for Publication Bias**

**Fig 1.** Funnel plot for total cholesterol in pre-eclampsia and normotensive pregnant women

**Fig 2.** Funnel plot for triglycerides in pre-eclampsia and normotensive pregnant women

**Fig 3.** Funnel plot for HDL-cholesterol in pre-eclampsia and normotensive pregnant women

**Fig 4.** Funnel plot for LDL-cholesterol in pre-eclampsia and normotensive pregnant women

**Fig 5.** Funnel plot for VLDL-cholesterol in pre-eclampsia and normotensive pregnant women
