# Supplementary material for: Maternal lipid profile and risk of pre-eclampsia in African pregnant women: A systematic review and meta-analysis
Source: PLoS One. 2020 Dec 23;15(12):e0243538. doi: 10.1371/journal.pone.0243538 (PMC7757810; doi:10.1371/journal.pone.0243538)
Supplement: S4 File — (DOCX) [file pone.0243538.s006.docx]

**Search strategy of lipid profiles in Pre-eclamptic African women**

| **S. No** | **Web site** | **Search terms** | **Result** |
| --- | --- | --- | --- |
| 1 | PubMed | ((((((("Lipids"[Mesh]) OR "Triglycerides"[Mesh]) OR "Cholesterol, HDL"[Mesh]) OR "Cholesterol"[Mesh]) OR "Cholesterol, VLDL"[Mesh]) OR "Cholesterol, LDL"[Mesh]) AND "Pre-Eclampsia"[Mesh]) AND "Africa"[Mesh] | 162 |
| 2 | Hinari | - Associations of lipid profiles with pre-eclampsia in Africa. - Association of total cholesterol, triglyceride, LDL-cholesterol, HDL-cholesterol and VLDL-cholesterol with Pre-eclampsia in African pregnant women. - Association of total cholesterol, triglyceride, LDL-cholesterol, HDL-cholesterol and VLDL-cholesterol with Pregnancy induced hypertension in Africa. | 241 |
| 3 | Google scholar | - Associations of lipid profiles with pre-eclampsia in Africa. - Association of total cholesterol, triglyceride, LDL-cholesterol, HDL-cholesterol and VLDL-cholesterol with Pre-eclampsia in African pregnant women. - Association of total cholesterol, triglyceride, LDL-cholesterol, HDL-cholesterol and VLDL-cholesterol with Pregnancy induced hypertension in Africa. | 43 |
| 4 | African Journals Online | - Associations of lipid profiles with pre-eclampsia in Africa. - Association of total cholesterol, triglyceride, LDL-cholesterol, HDL-cholesterol and VLDL-cholesterol with Pre-eclampsia in African pregnant women. - Association of total cholesterol, triglyceride, LDL-cholesterol, HDL-cholesterol and VLDL-cholesterol with Pregnancy induced hypertension in Africa. | 91 |

NB: These search terms used separately or in combination. Additionally, list of African countries were used for searching relevant articles
